# The Yeast Oxysterol Binding Protein Kes1 Maintains Sphingolipid Levels

**DOI:** 10.1371/journal.pone.0060485

**Published:** 2013-04-04

**Authors:** Marissa A. LeBlanc, Gregory D. Fairn, Sarah B. Russo, Ola Czyz, Vanina Zaremberg, L. Ashley Cowart, Christopher R. McMaster

**Affiliations:** 1 Department of Pathology, Dalhousie University, Halifax, Nova Scotia, Canada; 2 Keenan Research Centre of the Li Ka Shing Knowledge Institute, St. Michael's Hospital, Toronto, Ontario, Canada; 3 Department of Biochemistry and Molecular Biology, Medical University of South Carolina, Charleston, South Carolina, United States of America; 4 Department of Biology, University of Calgary, Calgary, Alberta, Canada; 5 Ralph H. Johnson Veterans Affairs Medical Center, Charleston, South Carolina, United States of America; 6 Department of Pharmacology, Dalhousie University, Halifax, Nova Scotia, Canada; Stony Brook University, United States of America

## Abstract

The oxysterol binding protein family are amphitropic proteins that bind oxysterols, sterols, and possibly phosphoinositides, in a conserved binding pocket. The *Saccharomyces cerevisiae* oxysterol binding protein family member Kes1 (also known as Osh4) also binds phosphoinositides on a distinct surface of the protein from the conserved binding pocket. In this study, we determine that the oxysterol binding protein family member Kes1 is required to maintain the ratio of complex sphingolipids and levels of ceramide, sphingosine-phosphate and sphingosine. This inability to maintain normal sphingolipid homeostasis resulted in misdistribution of Pma1, a protein that requires normal sphingolipid synthesis to occur to partition into membrane rafts at the Golgi for its trafficking to the plasma membrane.

## Introduction

Numerous regulatory mechanisms ensure proper levels of lipids within cells and organelles, with loss of control leading to various disease states. Glycerophospholipid and sterol synthesis occurs in the endoplasmic reticulum (ER), as do the first steps in the synthesis of sphingolipids. All three classes of lipids are transported to the Golgi apparatus where phospholipids can be further modified, and also serve as substrates for the final steps in the synthesis of sphingolipids. Lipid composition changes throughout the secretory pathway from a low concentration of sterols and sphingolipids in the ER with progressively higher levels in the Golgi and ultimately the plasma membrane. Membrane nanodomains or “rafts” formed by sphingolipids, sterols, saturated glycerophospholipids, and specific proteins have been proposed to be involved in the generation of this lipid gradient between the Golgi and the plasma membrane [1]. The processes integrating lipid metabolism with the formation of lipid rafts in the Golgi, and their subsequent transport out of this organelle are unclear. Lipid-binding proteins have been proposed to coordinate lipid metabolism with vesicular trafficking [2].

Yeast Osh proteins are part of an enigmatic class of lipid-binding proteins found throughout *Eukarya* united by a β-barrel structure that binds sterols, oxysterols, and phosphoinositides (PIPs) [3], [4]. The disruption of any six *OSH* genes in *Saccharomcyes cerevisiae* has minimal to mild affects on cellular growth [5]. However, inactivation of all seven *OSH* genes results in a substantial decrease in growth, indicating that Osh proteins most likely share an overlapping essential function [5]. Kes1 (Osh4) is the most well studied member of this family and numerous genetic and cell biology studies suggest Kes1 inhibits vesicular trafficking at the *trans*-Golgi. The mechanism is not clear, although it is known that Kes1 inhibits vesicular trafficking in a phosphatidylinositol (PI) 4-phosphate (PI-4P) dependent manner [6], [7], [8], [9], [10], [11], [12]. *In vitro*, Kes1 and other Osh proteins have been demonstrated to transfer sterols between membranes and a role for these proteins as direct non-vesicular sterol transporters has been proposed [9], [11]. Recent *in vivo* evidence is at odds with a role for Osh proteins in non-vesicular trafficking of sterols as cells diminished for function of all seven yeast Osh proteins displayed minimal defects in the intracellular transport of sterols [13], [14].

In this study, we reveal that Kes1 is required for maintenance of the normal ratio of complex sphingolipids and for maintenance of proper levels of sphingosine, sphingosine-phosphate, and ceramides. In the absence of Kes1 function, the localization of Pma1, a plasma membrane proton transporter whose trafficking from the Golgi to the plasma membrane requires sphingolipid synthesis, is compromised. We suggest that oxysterol binding protein superfamily members integrate sphingolipid metabolism with vesicular trafficking events.

## Materials and Methods

### Yeast strains and media

Rich medium was yeast extract protein dextrose (YEPD, 1% bacto-yeast extract, 2% bacto-peptone, 2% dextrose). Minimal medium was synthetic complete (SC, 0.67% bacto-yeast nitrogen base without amino acids, 2% dextrose, and nutrients as described). The *S. cerevisiae* strains used in this study are listed in Table 1. The CMY306 strain was constructed using standard molecular and yeast genetic methods whereby the *KES1* gene was inactivated in the SEY6210-*PMA1*-dsRFP strain. The *kes1*Δ::*HIS3* DNA region was amplified by polymerase chain reaction (PCR) from cells of strain CMY136 using primers hybridizing 500 base pairs (bp) upstream and downstream of the *KES1* open reading frame (ORF). The linear PCR product was transformed into SEY6210-*PMA1*-RFP cells and His^+^ transformants were screened by PCR of genomic DNA using primers hybridizing 600 bp upstream and downstream of the *KES1* ORF for confirmation of *kes1*Δ::*HIS3*.

**Table 1:** Yeast strains used in this study.

Yeast Strain	Genotype	Source
BY4741	*MAT*a *his3*Δ*1 leu2*Δ*0 met15*Δ*0 ura3*Δ*0*	Euroscarf
*kes1*Δ	BY4741 *kes1*Δ::*KanMx4*	Euroscarf
SEY6210	*MATα leu2-3, 112 ura3-52 his3-100 trp1-901 lys2-801 suc2-179*	Emr lab [24]
SEY6210-*PMA1*-dsRFP	SEY6210 *PMA1-dsRFP::KANMX4*	McMaster lab [19]
CMY136	*MATα sec14^ts^ ura3 his3 trp1 leu2 GAL+ kes1*Δ*::HIS3*	McMaster lab [7]
CMY306	SEY6210 *PMA1-dsRFP kes1*Δ*::HIS3*	This study

### Analysis of sphingolipids

For steady-state labeling cells were grown overnight to saturation. Cells were then seeded into low-density cultures and grown for at least six generations in the presence of 5 µCi/ml [^3^H]inositol. Aliquots of 5·10^7^ cells per point were harvested and pipetted into five volumes of ice-cold 10% tricholoroacetic acid solution. Lipids were extracted and resolved by thin layer chromatography in chloroform:methanol:NH_4_OH (9∶7∶2). Bands were visualized by exposure X-ray film, scraped into vials, and quantified by liquid scintillation counting. Liquid chromatography-mass spectrometry sphingolipid measurements were performed as described [15].

### Live cell imaging

Live cells were observed using a Zeiss Axiovert 200 M microscope fitted with a plan-neofluor 100× oil immersion lens. Images were captured using a Zeiss Axio Cam HR using Axiovision 4.5 software. Cells were visualized using differential interference contrast (DIC) and filters for rhodomine (for red fluorescent protein (RFP).

## Results

### The oxysterol binding protein Kes1 regulates complex sphingolipid metabolism

**Figure 1:**
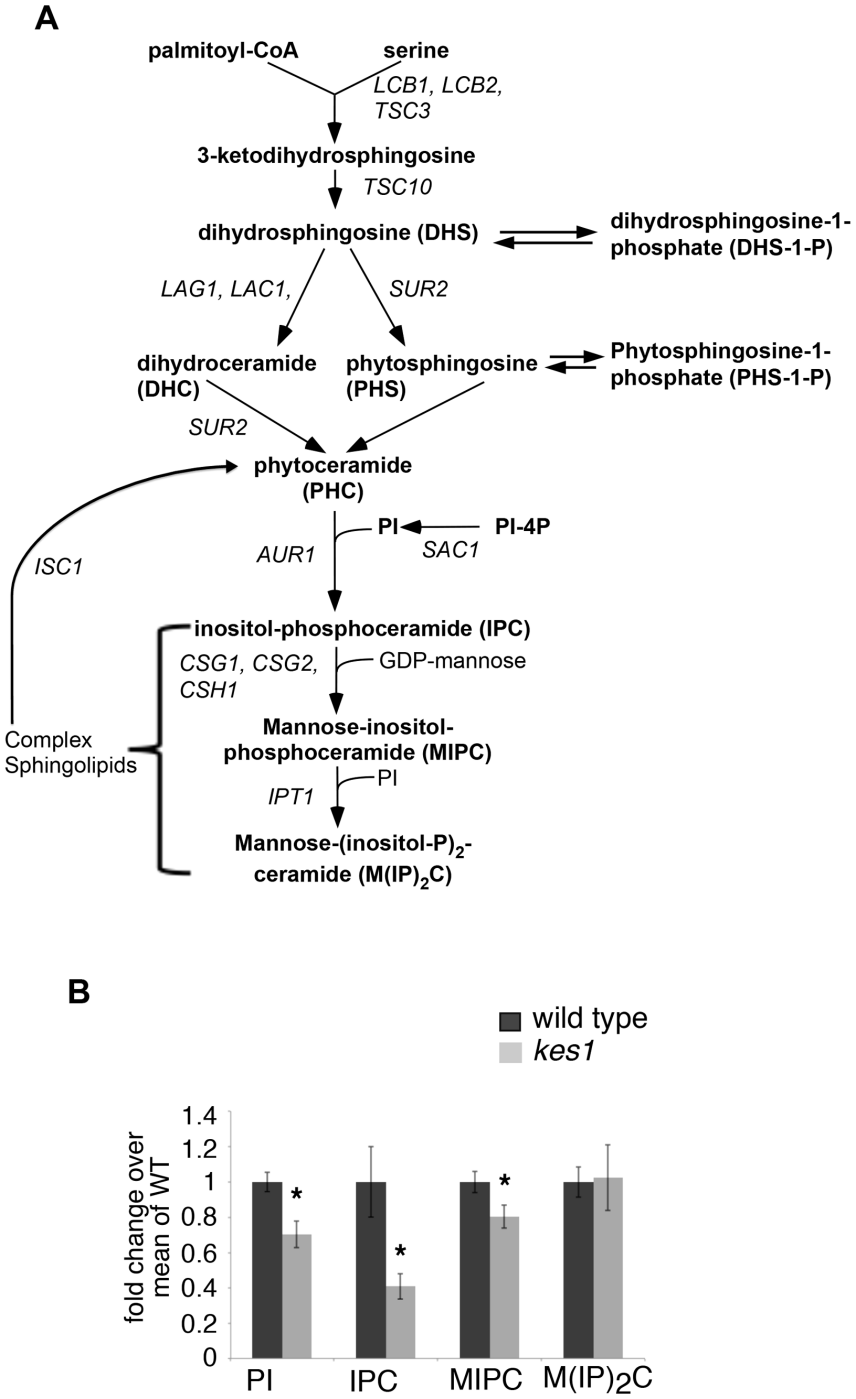
Kes1 enhances complex sphingolipid metabolism. (A) Phosphoinositides plays a central role in the synthesis of sphingolipids in yeast. Gene names are shown in italics. Metabolic intermediates and complex sphingolipids are shown in bold font. Inactivation of *KES1* inhibits the synthesis of sphingolipids. (B) Cells were grown to late logarithmic phase and inoculated into medium containing *myo*-[^3^H]inositol, grown to mid-logarithmic phase and lipids were extracted and resolved by thin layer chromatography. Radioactivity of the resolved lipids was measured for PI, IPC, MIPC and M(IP)_2_C. Values were normalized to cell number and are presented as fold-change over wild type. Data represent mean +/− SE of four independent experiments performed in triplicate. *P<0.05 respective to control.

PI serves as the donor of head groups of the synthesis of complex sphingolipids in *S. cerevisiae* (Fig. 1A). The oxysterol binding protein Kes1 has been recently determined to enhance the conversion of PI-4P to PI by the PI-4P phosphatase Sac1 [10]. This prompted us to investigate if in cells with an inactivated *KES1* gene there were altered levels of sphingolipids. We labeled cells with radiolabeled inositol to steady state and determined its incorporation into the metabolites of the sphingolipid biosynthetic pathway of *S. cerevisiae* to determine relative levels of complex sphingolipids. We observed that in cells with an inactivated *KES1* gene there was a 30% decrease in radiolabel incorporation into PI, a 60% decrease into the sphingolipid inositol-phosphoceramide (IPC), and a 20% decrease in mannosylinositolphosphorylceramide (MIPC), while labeling of mannosyldiinositolphosphorylceramide M(IP)_2_C was unchanged (Fig. 1B). These data indicate that Kes1 is required to maintain a normal cellular sphingolipid composition.

### The levels of sphingoid bases and ceramides are altered by Kes1

**Figure 2:**
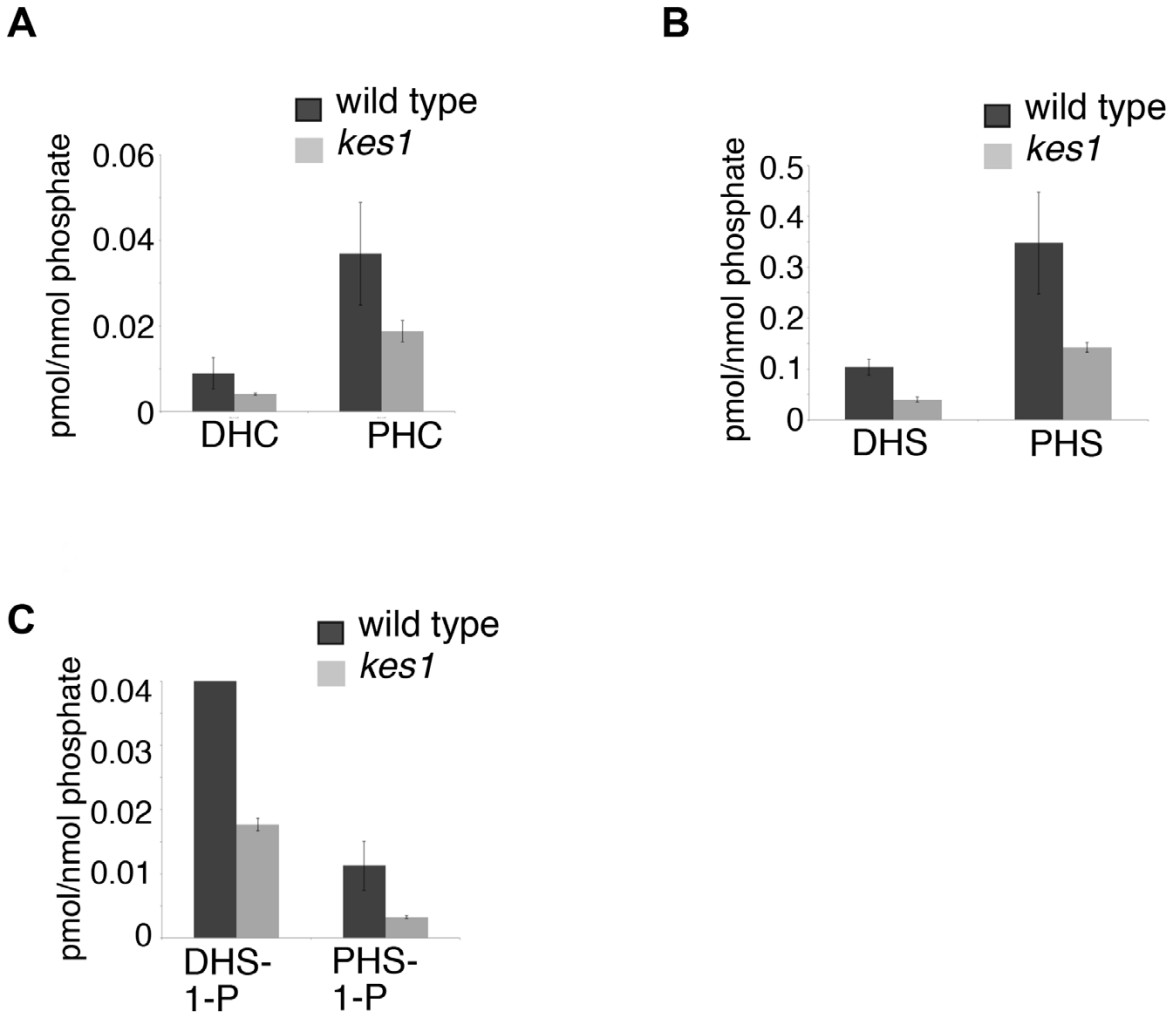
Kes1 affects sphingoid base and ceramide levels. Sphingolipids were extracted and measured by liquid chromatography-mass spectrometry and normalized to total inorganic phosphate. Total lipid levels representing all chain lengths are given for (A) dihydroceramide (DHC) and phytoceramide (PHC), (B) dihydrosphingosine (DHS) and phytosphingosine (PHS) and (C) dihydrosphingosine-1-phosphate (DHS-1-P) and phytosphingosine-1-phosphate (PHS-1-P). Data are expressed as a mean +/− SE of a minimum of three separate experiments.

**Figure 3:**
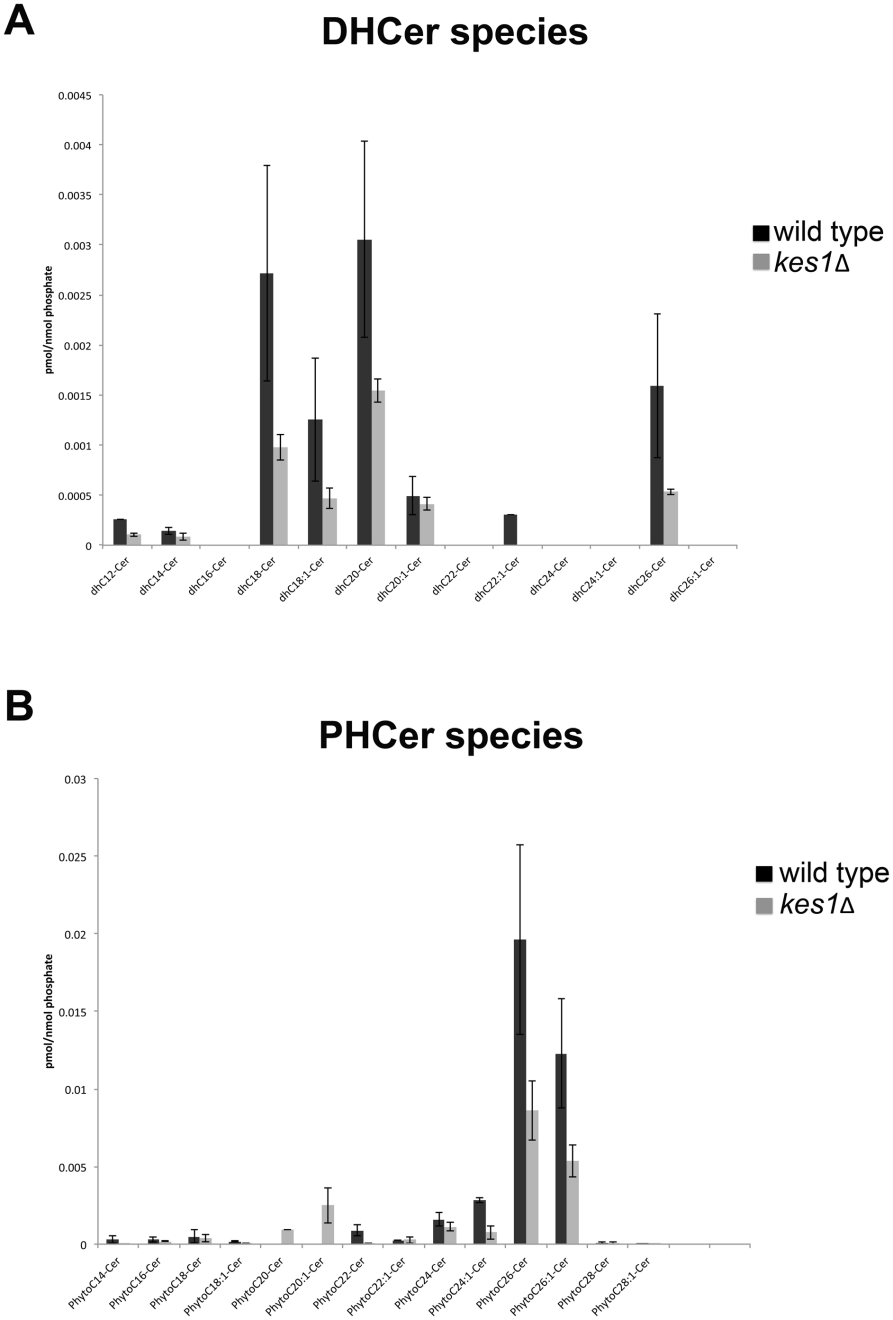
Ceramide species is unchanged in cells lacking Kes1. Lipids were extracted from cells and (A) dihydroceramide (DHCer) and (B) phytoceramide (PHCer) measured by liquid chromatography-mass spectrometry and normalized to total inorganic phosphate.

There is interconversion between complex sphingolipids, ceramides, and sphingoid bases with each having important cell signaling roles. We analyzed the level of sphingoid bases and ceramides using mass spectrometry to determine if Kes1 affected their levels and fatty acid composition. Inactivation of *KES1* resulted in a 50% reduction in the level of sphingosines, sphingosine- phosphates and ceramides (Fig. 2A–C). An analysis of the composition of fatty acids within yeast ceramides revealed an overall decrease in all acyl species (Fig. 3). Kes1 is required to maintain sphingoid base, ceramide and complex sphingolipid levels.

### Kes1 is required for proper localization of the lipid raft protein Pma1

**Figure 4:**
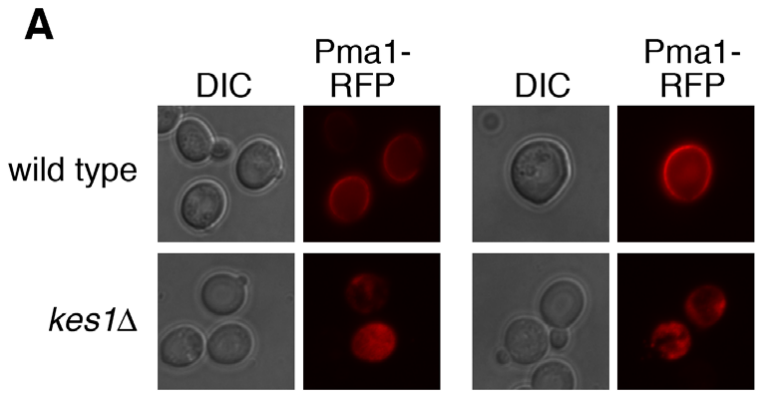
Kes1 regulates Pma1 localization. SEY6210-*PMA1*-dsRFP (wild type) and SEY6210-*PMA1*-dsRFP *kes1*Δ (CMY306) cells were grown to mid-logarithmic phase in SC medium at 25°C and visualized using a RFP filter. For quantification, 150 and 120 cells were counted for wild type and *kes1*Δ cells, respectively, over three different experiments to determine the percentage of cells with Pma1-RFP intracellular accumulation (6% for wild type and 81% for *kes1*Δ). Representative images are shown.

We assessed if the altered sphingolipid levels in cells lacking *KES1* had a physiological effect by determining if there was altered localization of a Pma1-RFP fusion protein. Normal sphingolipid synthesis is required to properly partition this endogenous membrane raft associated protein into lipid rafts at the Golgi for subsequent delivery to the plasma membrane [16]. As expected, in wild type cells Pma1-RFP was at the plasma membrane while in cell with an inactivated *KES1* gene the majority of Pma1-RFP was intracellular (Fig. 4). Quantification of this distribution determined that Pma1-RFP was primarily intracellular in 81% of cells with an inactivated *KES1* gene, compared to 6% in wild type cells. This implies that the defects in sphingolipid levels in *kes1*Δ cells affect trafficking and localization of Pma1p.

## Discussion

A major finding from this study is that Kes1 regulates the levels of complex sphingolipids, ceramides, sphingosine-phosphate, and sphingosine. The ratio of complex sphingolipids was skewed in cells with an inactivated *KES1* gene such that those upstream in this section of the sphingolipid biosynthetic pathway were decreased the most. This is consistent with the recent discovery that Kes1 can activate the PI-4P phosphatase Sac1 to produce PI [10], with the PI produced being preferentially used for the synthesis of complex sphingolipids [17]. Inactivation of the *KES1* gene did not affect the overall fatty acid composition within ceramide species, however, an unanticipated finding was that Kes1 was required to maintain appropriate levels of sphingosines, sphingosine-phosphates, and ceramides. A physiological effect upon inactivation of the *KES1* gene was abnormal distribution of the endogenous lipid raft localized protein Pma1, with Pma1 found primarily intracellularly rather than at the plasma membrane.

Complex sphingolipid synthesis takes place in the Golgi while sphingoid base and ceramide synthesis occurs in the ER. Proper Pma1 localization can be affected by defects in sphingolipid synthesis [16], [18], or by perturbing lipid raft composition at the plasma membrane [19]. It is unclear from the current study which of these is occurring. However, we had previously determined that Kes1 localizes to the Golgi [8]. This suggests that partitioning of Pma1 into appropriate vesicles for trafficking from the Golgi to the plasma membrane is likely being affected. Indeed, results from a genome-wide screen demonstrated that yeast with an inactivated *KES1* gene, or defective in sterol and sphingolipid synthesis, were unable to traffic Fus-Mid-GFP. Fus-Mid-GFP is an engineered protein composed of the extracellular region of Fus1p fused to the transmembrane domain and cytoplasmic tail of Mid2p followed by GFP. Fus-Mid-GFP has to associate with lipid rafts to exit the Golgi and to traffic to the plasma membrane [20]. Previous data showed that inactivation of the *KES1* gene did not alter the secretion of invertase [20], supporting a notion that Kes1 regulates specific vesicular pathways out of the *trans*-Golgi. We also noted that inactivation of *KES1* did not affect FM4-64 trafficking from the plasma membrane to the vacuole (data not shown) consistent with this notion.

Large scale genome-wide association studies have determined that inactivation of the *KES1* gene is synthetic sick with genes of the sterol biosynthetic pathway in yeast, but not those of the sphingolipid pathway [21]. This is consistent with Kes1 affecting the level of sphingolipid synthesis, a process that works in parallel with the sterol synthesis pathway for a shared downstream function. This interpretation is also in agreement with a study where it was identified that Kes1 inhibits phospholipid flippase activity at the *trans*-Golgi, likely indirectly, to relax membrane curvature to establish an ideal environment for sterol-rich rafts and vesicles to form [12]. The above observations combined with the data here that determined that Kes1 is necessary for normal sphingolipid levels and distribution of the lipid raft protein Pma1, imply that Kes1 may aid in assembly of membrane rafts at the Golgi for subsequent trafficking of rafts and their cargo to the plasma membrane.

Interestingly, in contrast to that observed for inactivation of the *KES1* gene where we observed a decrease in the level of sphingosine, sphingosine-phosphate, and ceramides, inactivation of *SAC1* resulted in an elevation of these lipids [17]. Sac1 localizes to both the Golgi and ER while Kes1 has only been reported to associate with the Golgi. Sphingoid bases are synthesized in the ER where Sac1 was reported to be part of the SPOTS complex comprised of the first enzyme in the sphingoid base pathway serine palmitoyltransferase, Tsc3 (a regulatory subunit of serine palmitoyltransferase), and Orm1/2 [22], [23]. Similar to the inactivation of *SAC1*, the loss of Orm function results in an accumulation of sphingoid bases. In addition, deletion of the *ORM1* and *ORM2* genes is synthetic lethal with deletion of *SAC1* consistent with their regulating sphingoid base synthesis as part of a protein complex. Unlike Sac1, Kes1 is not found at the ER and is not part of the SPOTS complex. How Kes1 regulates sphingoid base levels will require further work.
